# Surgical Treatment of Diabetic Foot Ulcers Complicated by Osteomyelitis with Gentamicin-Loaded Calcium Sulphate-Hydroxyapatite Biocomposite

**DOI:** 10.3390/jcm10020371

**Published:** 2021-01-19

**Authors:** Kor H. Hutting, Wouter B. aan de Stegge, Jaap J. van Netten, Wouter A. ten Cate, Luuk Smeets, Gijs M. J. M. Welten, Dick M. Scharn, Jean-Paul P. M. de Vries, Jeff G. van Baal

**Affiliations:** 1 Department of Surgery, Hospital Group Twente, Zilvermeeuw 1, 7609 PP Almelo, Overijssel, The Netherlands; j.j.vannetten@amsterdamumc.nl (J.J.v.N.); W.tCate@zgt.nl (W.A.t.C.); j.vbaal@zgt.nl (J.G.v.B.); 2 Division of Vascular Surgery, Department of Surgery, University Medical Center Groningen, Hanzeplein 1, 9713 GZ Groningen, Groningen, The Netherlands; w.b.aandestegge@amsterdamumc.nl (W.B.a.d.S.); j.p.p.m.de.vries@umcg.nl (J.-P.P.M.d.V.); 3 Department of Rehabilitation, University of Amsterdam, Amsterdam UMC, Amsterdam Movement Sciences, Meibergdreef 9, 1105 AZ Amsterdam, Noord-Holland, The Netherlands; 4 Department of Surgery, Rijnstate Hospital, Wagnerlaan 55, 6815 AD Arnhem, Gelderland, The Netherlands; LSmeets@rijnstate.nl; 5 Department of Surgery, Franciscus Gasthuis & Vlietland, Kleiweg 500, 3045 PM Rotterdam, Zuid-Holland, The Netherlands; g.welten@franciscus.nl; 6 Department of Surgery, Slingeland Hospital, Kruisbergseweg 25, 7009 BL Doetinchem, Gelderland, The Netherlands; d.scharn@slingeland.nl; 7 Welsh Wound Innovation Centre, Rhodfa Marics, Ynysmaerdy, Pontyclun CF72 8UX, Wales, UK

**Keywords:** diabetes mellitus, foot ulcers, foot infections, gentamicin-loaded calcium sulphate-hydroxyapatite biocomposite, osteomyelitis, surgery

## Abstract

Diabetic foot ulcers, complicated by osteomyelitis, can be treated by surgical resection, dead space filling with gentamicin-loaded calcium sulphate-hydroxyapatite (CaS-HA) biocomposite, and closure of soft tissues and skin. To assess the feasibility of this treatment regimen, we conducted a multicenter retrospective cohort study of patients after failed conventional treatments. From 13 hospitals we included 64 patients with forefoot (*n* = 41 (64%)), midfoot (*n* = 14 (22%)), or hindfoot (*n* = 9 (14%)) ulcers complicated by osteomyelitis. Median follow-up was 43 (interquartile range, 20–61) weeks. We observed wound healing in 54 patients (84%) and treatment success (wound healing without ulcer recurrence) in 42 patients (66%). Treatment failures (no wound healing or ulcer recurrence) led to minor amputations in four patients (6%) and major amputations in seven patients (11%). Factors associated with treatment failures in univariable Cox regression analysis were gentamicin-resistant osteomyelitis (hazard ratio (HR), 3.847; 95%-confidence interval (CI), 1.065–13.899), hindfoot ulcers (HR, 3.624; 95%-CI, 1.187–11.060) and surgical procedures with gentamicin-loaded CaS-HA biocomposite that involved minor amputations (HR, 3.965; 95%-CI, 1.608–9.777). In this study of patients with diabetic foot ulcers, complicated by osteomyelitis, surgical treatment with gentamicin-loaded CaS-HA biocomposite was feasible and successful in 66% of patients. A prospective trial of this treatment regimen, based on a uniform treatment protocol, is required.

## 1. Introduction

In people with diabetes mellitus, foot ulcers are a major complication, with a lifetime prevalence of 19–34% [1]. An estimated 18.6 million people are currently affected by diabetic foot ulcers [2]. Approximately 60% of diabetic foot ulcers are infected at presentation, and 40% of the noninfected diabetic foot ulcers become infected before healing [3,4]. Infected diabetic foot ulcers increase morbidity and are the most common cause of diabetes-related hospitalizations and lower extremity amputations [5,6]. Approximately 20% of infected diabetic foot ulcers are complicated by osteomyelitis, which is defined as infection of the bone with involvement of bone marrow [7,8]. In patients with diabetic foot ulcers complicated by osteomyelitis (DFO), ulcer healing is impaired and the amputation risk is increased [9,10,11].

Current treatment regimens for patients with DFO consist of standard foot ulcer management (offloading, restoration of tissue perfusion, local ulcer care with debridement and wound dressings), optimization of glycemic control with antibiotic therapy, and, when required, surgical resection of necrotic and infected soft tissues and necrotic bone [12,13,14,15]. These treatments may result in wound healing in 78% to 86% of patients [16,17,18]. However, surgical resection requires minor amputations (distal to the ankle) in up to 40% of patients. In 6% to 23% of these patients, successive minor amputations on more proximal levels are required due to treatment failures, and up to 9% of these patients end up undergoing a major amputation [16,17,18,19].

Several new treatment regimens are being developed to improve outcomes, with the aim of reducing amputation rates and preserving patient mobility. Surgical resection of macroscopically necrotic and infected soft tissues and necrotic bone with subsequent filling of the resultant void (the “dead space”) using a calcium sulphate-hydroxyapatite (CaS-HA) biocomposite bone graft substitute loaded with gentamicin, could be a promising new treatment regimen [20,21]. This treatment regimen requires surgical closure by primary closure or reconstructive techniques to prevent CaS-HA biocomposite leakage and to cover exposed bone [22,23,24]. Therefore, surgical procedures in this treatment regimen are often performed in one stage. Local release of gentamicin from the CaS-HA biocomposite leads to a high tissue concentration of gentamicin for several weeks that eradicates pathogens [25,26]. Furthermore, the CaS-HA biocomposite functions as an osteoconductive scaffold that supports bone augmentation and prevents bone resorption [20,22,27].

Promising results have been reported on this treatment regimen for DFO in three previous retrospective studies and one case report [23,24,28,29]. Uncomplicated wound healing is reported in 80% to 100% of these patients, and amputations are only reported after treatment failures [23,24,28,29]. However, these previous studies all have a retrospective design and lack details of treatments (e.g., antibiotic therapy or offloading) and other outcomes than postoperative wound healing (e.g., ulcer recurrence) [23,24,28,29]. Therefore, additional investigation is required to assess the feasibility of this treatment regimen.

Currently, the use of gentamicin-loaded CaS-HA biocomposite is not incorporated in (inter)national guidelines for the treatment of DFO [14,30,31]. However, several hospitals in the Netherlands have used gentamicin-loaded CaS-HA biocomposite to treat patients with DFO after failed conventional treatments. We conducted a multicenter, retrospective cohort study to investigate the feasibility of this treatment regimen for DFO after failed conventional treatments.

## 2. Experimental Section

We conducted a retrospective multicenter cohort study of patients treated between February 2017 and June 2019. Retrospective data collection was completed in December 2019. Dutch hospitals in which surgical treatments of DFO with gentamicin-loaded CaS-HA biocomposite were performed were contacted. The physicians (vascular surgeons and/or trauma surgeons) who performed the surgical treatments were assigned as local investigators. Patients provided written informed consent for data collection, analysis and storage. We performed all study procedures according to the Declaration of Helsinki and the Dutch Personal Data Protection Act. The Medical Ethics Committee Twente ruled this study exempt from formal ethical approval because of the retrospective observational design (METC Twente project K18-33).

We included patients with diabetes mellitus with one or more foot ulcers complicated by suspected or confirmed osteomyelitis who underwent a surgical procedure with gentamicin-loaded CaS-HA biocomposite (Cerament G™; BoneSupport, Lund, Sweden). We defined a foot ulcer as a discontinuation of the skin that minimally includes the epidermis and part of the dermis [8]. In accordance with the International Working Group on the Diabetic Foot (IWGDF) guidelines and the Dutch national guidelines, suspected osteomyelitis was defined as the presence of suggestive clinical signs (e.g., positive probe-to-bone test, exposed bone and/or intraosseous pus at intervention), elevated serum inflammatory markers and suggestive findings of osteomyelitis on imaging assessment (x-ray imaging, computed tomography, magnetic resonance imaging or radionuclide imaging) [14,30,32]. Confirmed osteomyelitis was defined as bone samples with cultures positive for microbiological pathogens [14,30,32].

Prerequisites for performing surgical procedures with gentamicin-loaded CaS-HA biocomposite were that the soft tissues and skin around the ulcer were adequate for closure of surgical wounds by primary closure or by reconstructive techniques. All patients were treated with gentamicin-loaded CaS-HA biocomposite as a last resort after conventional treatments of DFO failed. Failed conventional treatment was defined as a persistent foot ulcer with unresolved osteomyelitis after prolonged antibiotic therapy (>6 weeks [14]) or a persistent wound with unresolved osteomyelitis after surgical resection of DFO or minor amputation with adjuvant antibiotic therapy (approximately 1 week [14]). Minor amputation was defined as any resection through or distal to the ankle, in accordance with the IWGDF definition [8]. Osteomyelitis was in these cases diagnosed as described above. Only the first procedure was included if patients underwent multiple treatments with gentamicin-loaded CaS-HA biocomposite.

We excluded patients with severe chronic limb ischemia, irrespective of preoperative revascularization, because of the low probability of postoperative wound healing [33]. Severe chronic limb ischemia was defined in accordance with the wound, ischemia and foot infection classification as an ankle-brachial index ≤0.39, an ankle systolic pressure <50 mmHg, a systolic toe pressure <30 mmHg or a transcutaneous oxygen pressure <30 mmHg [33].

**Figure 1:**
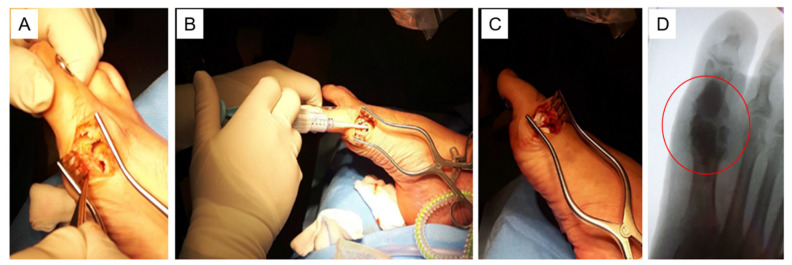
Surgical treatment with gentamicin-loaded calcium sulphate-hydroxyapatite (CaS-HA) biocomposite of a diabetic foot ulcer, complicated by osteomyelitis, at the medial aspect of the first metatarsophalangeal joint. (**A**) After surgical excision of the foot ulcer, macroscopic necrotic bone and necrotic and infected soft tissues are resected. (**B**) The dead space is irrigated with saline solution, and gentamicin-loaded CaS-HA biocomposite is injected. (**C**) The dead space is filled with gentamicin-loaded CaS-HA biocomposite. (**D**) In this dorsal-plantar x-ray image of the foot, the gentamicin-loaded CaS-HA biocomposite is visible as a density in the distal part of the metatarsal and the proximal phalanx (encircled).

When this study was conducted, no uniform treatment protocol existed for the surgical treatment of DFO with gentamicin-loaded CaS-HA biocomposite. The surgical procedures with gentamicin-loaded CaS-HA biocomposite generally consisted of the following steps: After excision of one or more foot ulcers, macroscopic necrotic bone and necrotic and infected soft tissues were resected (Figure 1A). In most cases, surgeons obtained one or more bone samples for examination of microbiological pathogens. The remaining dead space was irrigated with saline solution and filled with CaS-HA biocomposite loaded with gentamicin (17.5 mg/mL) via injection or as pellets (Figure 1B,C). Guidance using x-ray imaging was used at the surgeons’ discretion. 

After the dead space was filled with gentamicin-loaded CaS-HA biocomposite, surgical wounds were closed by primary closure or reconstructive techniques (e.g., local transposition flaps). In certain patients with forefoot or midfoot DFO, complete resection of necrotic bone as part of the surgical procedure with gentamicin-loaded CaS-HA biocomposite resulted in a transmetatarsal amputation. These amputations were performed on a level as distally as possible, with the aim of preventing subsequent extensive proximal amputations.

After resection, the intramedullary canals of the residual metatarsals were filled with gentamicin-loaded CaS-HA biocomposite, followed by surgical closure of the amputation wounds. Temporary or definitive fixation (e.g., external fixation or Kirschner wires) methods were also required in certain patients because of biomechanical instability after bone resection. After resection of a joint, the residual dead space was filled with gentamicin-loaded CaS-HA biocomposite to create a semi-rigid or rigid arthrodesis, irrespective of fixation methods used.

Decisions regarding postoperative antibiotic therapy were made independently by the treating physicians in the absence of a uniform treatment protocol. In general, postoperative antibiotic therapy was only administered to patients with extensive DFO in whom adequate surgical resection of all necrotic and infected tissues was difficult. Postoperative offloading was advised in all patients until postoperative wound healing was observed. Again, lack of a uniform treatment protocol resulted in individual decisions of treating physicians regarding the use of offloading devices and postoperative wound care (e.g., bandages).

In the participating centers, local investigators selected patients according to the inclusion criteria and retrospectively collected data from electronic health record systems which were registered in a secured database (OpenClinica LLC, Version 3.13, Waltham, MA, USA). Data regarding demographics, comorbidities, index ulcers and affected feet were collected. We defined the index ulcer as the clinically most important foot ulcer with the clearest association to the underlying osteomyelitis as judged clinically and based on imaging findings. Index ulcers located around the metatarsals, the phalanges and associated soft tissues were classified as “forefoot index ulcers”, index ulcers located around the cuboid, navicular, cuneiform bones and associated soft tissues as “midfoot index ulcers,” and index ulcers around the talus, calcaneum and associated soft tissues as “hindfoot index ulcers” [8]. Additionally, we classified index ulcers according to the “Site, Ischemia, Neuropathy, Bacterial infection, Area, Depth” (SINBAD) classification [34].

We collected data of loss of protective sensation and deformities of the affected foot. Loss of protective sensation was defined as absence of pressure sensation of a 10-g monofilament [13]. We classified deformities as mild (pes cavus, hallux valgus, hallux limitus or hammer toes), moderate (hallux rigidus, claw toes or prominent metatarsal heads) or severe (Charcot neuroarthropathy-related deformity, previous ankle arthrodesis or previous partial calcanectomy) [35]. The deformity graded most severe determined the classification [35]. We also registered previous contralateral major amputations (defined as any resection proximal to the ankle in correspondence with the IWGDF definition) and previous ipsilateral minor amputations [8,35]. Moreover, we collected data regarding the surgical procedures and postoperative treatments. Furthermore, we collected data of the microbiological culture results of intraoperatively obtained bone samples, including gentamicin-resistance of pathogens which was investigated using the minimal inhibitory concentration breakpoints of the European Committee on Antimicrobial Sensitivity Testing (EUCAST) (Växjö, Sweden) [36]. 

Follow-up was completed until minor or major amputation after the initial surgery, repeated surgical resection of DFO, death or the last-mentioned consultation in the electronic health record system. We registered data regarding postoperative wound healing, ulcer recurrence, minor and major amputations, readmissions, reoperations, functional results and adverse events, including postoperative fractures, Charcot neuroarthropathy exacerbations and deaths. Wound healing was defined as macroscopic complete epithelialization after removal of abundant callus without drainage or requirement of wound dressings, maintained for a minimum of 2 weeks [8,15]. We defined ulcer recurrence as development of an ulcer on the same location as the index ulcer after initial postoperative wound healing irrespective of the presence of (ongoing) osteomyelitis. Functional results were classified as unable to mobilize weight-bearing, able to mobilize weight-bearing with a walking aid or able to mobilize weight-bearing without a walking aid. Information was also collected regarding footwear used at the final follow-up.

As primary outcomes, we assessed postoperative wound healing and ulcer recurrence. Treatment success was defined as uncomplicated wound healing without ulcer recurrence, and treatment failure was defined as presence of a persistent wound at final follow-up or ulcer recurrence after initial postoperative wound healing. A persistent wound was defined as absence of postoperative wound healing after primary closure or closure by reconstructive techniques. As secondary outcomes, we assessed minor and major amputations and functional results at final follow-up.

Categorical data are reported as numbers of patients with corresponding percentages and continuous data as means with standard deviations (SD) or medians with interquartile ranges (IQR) when nonparametric. We compared characteristics regarding demographics, comorbidities, index ulcers, affected feet, surgical procedures and postoperative treatments between patients with treatment success, patients with treatment failure due to persistent wounds, and patients with treatment failure due to ulcer recurrence after initial postoperative wound healing. These comparisons were made using one-way analyses of variances (ANOVA) for parametric continuous data, Kruskal–Wallis tests for nonparametric continuous data and Fisher exact tests for categorical data. All tests were performed two-sided (*α* = 0.05). Post hoc analyses were performed using a Bonferroni correction. 

A univariable Cox regression analysis was performed to investigate associations between treatment failures and all above-mentioned characteristics. We used a Kaplan–Meier curve to demonstrate treatment failures, in which patients with persistent wounds were indicated as having an event at day 1. SPSS 23.0 software (IBM, Armonk, NY, USA) was used for all statistical analyses.

## 3. Results

### 3.1. Patients and Procedures

**Figure 2:**
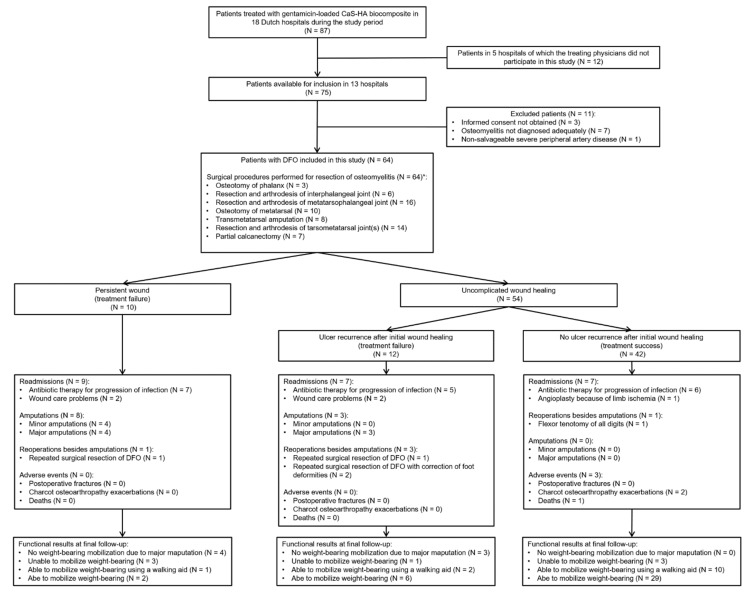
Study flowchart. CaS-HA: Calcium sulphate-hydroxyapatite. DFO: Diabetic foot osteomyelitis. * Details of the surgical procedures are listed in Appendix A: Table A1.

From 13 hospitals, we included 64 patients, of whom 49 (77%) had confirmed osteomyelitis and 15 (23%) had suspected osteomyelitis (Figure 2, Table 1). The surgical procedure with gentamicin-loaded CaS-HA biocomposite involved minor amputations in five patients (8%) with forefoot DFO and three patients (5%) with midfoot DFO (Table 1). Details of the surgical procedures are listed in Appendix A: Table A1.

### 3.2. Primary Outcomes

Median postoperative follow-up was 43 (IQR, 20–61) weeks (Table 1). We observed uncomplicated wound healing in 54 patients (84%) and a median time to wound healing of 9 (IQR, 5–16) weeks (Figure 2). Of 10 patients (16%) with persistent wounds, four underwent minor amputations during follow-up, four underwent major amputations, one underwent repeated surgical resection of DFO without antibiotic-loaded CaS-HA biocomposite, and one still had an ongoing wound at a final follow-up of 14 weeks (Figure 2). Recurrent foot ulcers were observed in 12 of 54 patients (22%) after initial postoperative wound healing, and the median time to ulcer recurrence was 24 (IQR, 16–46) weeks (Figure 2). Thus, the rate of treatment success was 66% (42 patients) overall (Figure 2).

Of 12 patients (19%) with ulcer recurrence, three underwent major amputations, three underwent repeated surgical resections of DFO without antibiotic-loaded CaS-HA biocomposite, three underwent successful conservative treatments by antibiotic therapy and offloading and three had ongoing ulcers at final follow-up (Figure 2). In patients with ulcer recurrence, the preoperative index ulcer lasted significantly longer compared with patients without ulcer recurrence (median, 37 (IQR, 21–79) weeks vs. median 18 (IQR, 10–43) weeks, respectively; *p* = 0.014) (Table 1). Furthermore, a significantly larger proportion of patients with persistent wounds (*n* = 4 (40%)) underwent surgical procedures with gentamicin-loaded CaS-HA biocomposite that involved minor amputations compared with patients with ulcer recurrence (*n* = 3 (25%)) or treatment success (*n* = 1 (2%); *p* = 0.004) (Table 1).

### 3.3. Secondary Outcomes

After a median follow-up of 8 (IQR, 5–23) weeks, minor amputations were performed because of treatment failures in four patients (6%), of whom two initially underwent surgical procedures with gentamicin-loaded CaS-HA biocomposite that involved minor amputations (Figure 2). Major amputations were performed after a median follow-up of 17 (IQR, 7–41) weeks in seven patients (11%), of whom five initially underwent surgical procedures with gentamicin-loaded CaS-HA biocomposite that involved minor amputations (Figure 2). At the final follow-up, 50 patients (78%) could mobilize weight-bearing, including 11 patients (17%) who had treatment failures (Figure 2). Of these 50 patients, 48 (96%) used custom-made or prefabricated therapeutic footwear, and two (4%) used prefabricated footwear at final follow-up. Three of 14 patients (22%) who were unable to mobilize weight-bearing had pre-existent incomplete paraplegia (Figure 2).

### 3.4. Treatment Failures

**Figure 3:**
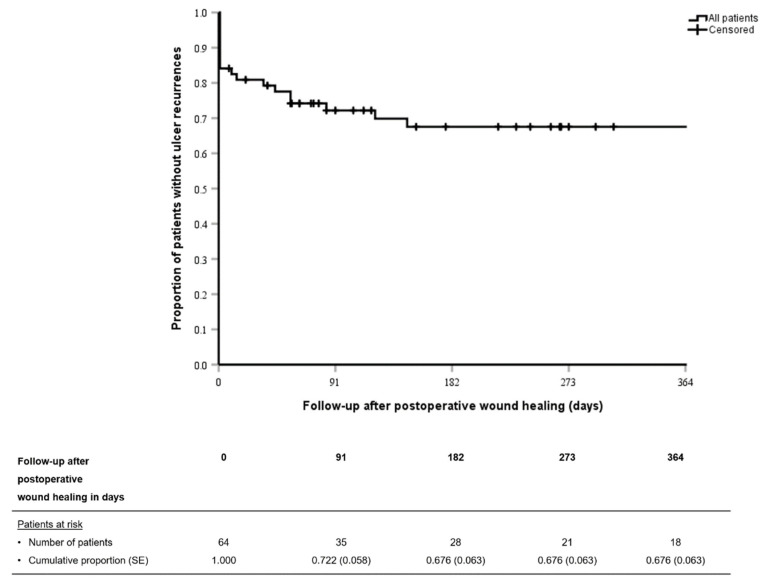
Kaplan–Meier curve demonstrating treatment failures (persistent wounds or ulcer recurrence after initial postoperative wound healing) of surgical treatments with gentamicin-loaded calcium sulphate-hydroxyapatite biocomposite of diabetic foot ulcers complicated by osteomyelitis. Patients with persistent wounds are demonstrated as having an event at day 1. SE: Standard error.

Patients with persistent wounds (defined as treatment failure at day one for time-based analyses) and patients with ulcer recurrence after initial postoperative wound healing are demonstrated in the Kaplan-Meier curve in Figure 3. In univariable Cox regression analysis, factors that were independently associated with treatment failure were gentamicin-resistant DFO, index ulcer location and surgical procedures with gentamicin-loaded CaS-HA biocomposite that involved a minor amputation (Table 2).

### 3.5. Microbiological Analysis 

**Figure 4:**
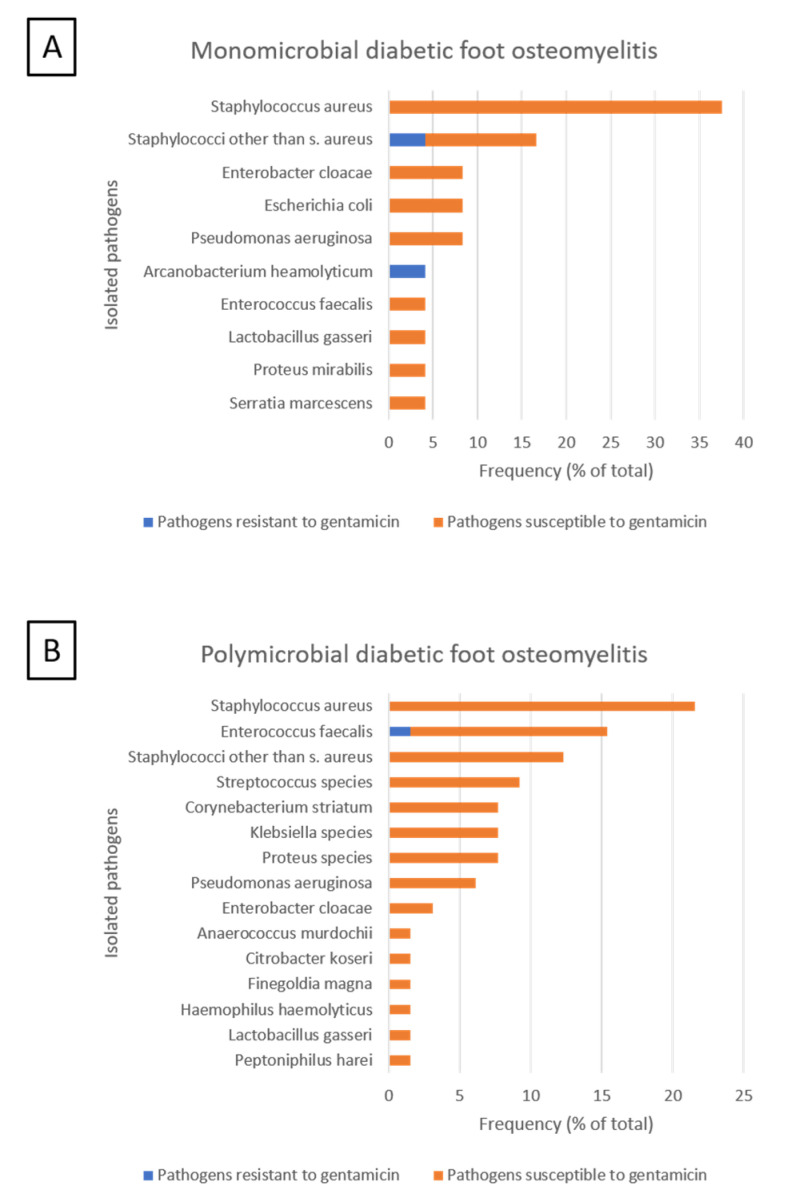
Pathogens isolated in bone samples of 49 patients with diabetic foot ulcers complicated by osteomyelitis. (**A**) Distribution of pathogens isolated in 16 patients with monomicrobial osteomyelitis. (**B**) Distribution of 87 pathogens isolated in 33 patients with polymicrobial osteomyelitis. Gentamicin-resistance of pathogens was based on the minimal inhibitory concentration breakpoints of the European Committee on Antimicrobial Sensitivity Testing (EUCAST).

Bone samples were obtained in 49 patients (76%). Microbiological analysis yielded a median of two (IQR, 1–3) pathogens per patient. Osteomyelitis was monomicrobial in 24 patients (38%) and polymicrobial in 25 patients (39%), with Staphylococcus aureus as the most frequently isolated pathogen in both groups (Table 1, Figure 4).

### 3.6. Postoperative Treatment

Postoperative antibiotic therapy was administered in 26 patients (41%) for a median of 3 (IQR, 2–6) weeks. Postoperative antibiotic therapy was administered to four patients (40%) without postoperative wound healing, which was not significantly (*p* = 0.930) different from patients with ulcer recurrence (*n* = 4 (33%)) or without ulcer recurrence (*n* = 18 (43%)). Postoperative offloading was performed in all patients for median 6 (IQR, 5–8) weeks by non-weight-bearing mobilization (*n* = 33 (52%)), nonremovable knee-high devices (*n* = 14 (22%)), removable knee-high devices (*n* = 2 (3%)) and removable ankle-high devices (*n* = 15 (3%)).

### 3.7. Adverse Events

Readmissions, reoperations and adverse events are listed in Figure 2. Charcot osteoarthropathy exacerbations were observed in two of 18 patients (11%) with Charcot osteoarthropathy during follow-up (Figure 2). After 23 weeks of follow-up, one patient with postoperative wound healing without ulcer recurrence died of cardiac disease. No other patients were lost to follow-up.

## 4. Discussion

In this multicenter, retrospective cohort study, we investigated the treatment of DFO with gentamicin-loaded CaS-HA biocomposite in patients where conventional treatment had failed. Treatment success was observed in 66% of patients during median 43 weeks of follow-up. Treatment failure due to a persistent wound or ulcer recurrence after initial postoperative wound healing was observed in 15% and 19% of patients, respectively. After treatment failure, minor and major amputations were required in 6% and 11% of patients, respectively. Furthermore, 78% of patients could mobilize weight-bearing at final follow-up, including 17% patients with treatment failures. These findings confirm results from previous studies and show that surgical treatment with gentamicin-loaded CaS-HA biocomposite is feasible for patients with DFO after failed conventional treatments [23,24,28,29].

Our findings correspond with previous publications of this treatment regimen regarding wound healing rates (details are listed in Table 3) [23,24,28,29]. However, there are important differences between our study and previous publications regarding locations of DFO, surgical procedures and postoperative treatments. First, contrary to previous studies in which patients with midfoot or hindfoot DFO were predominantly included, we mainly included patients with forefoot DFO [24,28,29].

Second, the surgical procedures with gentamicin-loaded CaS-HA biocomposite involved minor amputations in 13% of patients in our study, whereas these were only reported in one previous study in two of 70 patients [23]. This is probably the result of the high proportion of patients with forefoot DFO in our study, in whom complete surgical resection sometimes can only be performed by minor amputation.

Third, only 41% of patients in our study received postoperative antibiotic therapy, whereas all patients were administered postoperative antibiotic therapy for several weeks in previous studies [23,24,28,29]. This difference is probably caused by the lack of uniform treatment protocols. The observed success rate, obtained in a study population of which more than half was not treated by postoperative antibiotic therapy, could suggest that systemic antibiotic therapy is not indicated in all patients after treatment with gentamicin-loaded CaS-HA biocomposite. However, further investigation of the role of systemic antibiotic therapy in this treatment regimen is required before recommendations can be made.

Finally, vacuum-assisted closure of surgical wounds was not performed in our study, whereas this was performed in up to 50% of patients in previous studies [23,24]. These differences limit comparisons of our study with previous publications, and indicate that uniform protocols are needed regarding patient selection, surgical procedures and postoperative treatment for this treatment regimen.

The ultimate treatment goal in people with DFO is to become ulcer-free. Therefore, our definition of treatment failure includes ulcer recurrence. Because only postoperative wound healing was considered in previous studies of this treatment regimen, reported success rates might be overestimated [23,24,29]. We postulate that ulcer recurrence should be included in the definitions of treatment failure in future studies. Furthermore, we recommend reporting details regarding offloading and other ulcer prevention strategies in future treatment protocols, because inadequate offloading is one of the possible causes for ulcer recurrence [35].

In this study, we explored potential risk factors for treatment failures. In a univariable analysis, gentamicin-resistant DFO, hindfoot DFO and surgical procedures with gentamicin-loaded CaS-HA biocomposite that involved minor amputations were associated with treatment failures. These results should be interpreted with caution given the high confidence intervals and the small sample size with a limited number of events. However, since these potential associations might be clinically relevant, they should be investigated further. These investigations should include postoperative offloading, since the potential association between hindfoot DFO and treatment failures could be the result of offloading difficulties [38,39,40].

Amputations are frequently performed in patients with DFO after unsuccessful conventional treatments [10,11]. Minor and major amputations were performed in 6% and 11% of patients, respectively, after failed treatments with gentamicin-loaded CaS-HA biocomposite in our study. These rates are higher than the rates of minor (0–3%) and major (7–9%) amputations reported in previous studies of this treatment regimen [23,29]. This is probably caused by the inclusion of patients after failed conventional treatments, who had no options left after failure of treatment with gentamicin-loaded CaS-HA biocomposite. However, considering the selection of patients after failed conventional treatments, the observed rates of minor and major amputations were lower than expected. These findings warrant further research into this new treatment regimen, in which amputations performed after treatment failures should also be investigated.

Our study has several limitations. First, the retrospective design imposes a risk of bias. Second, the absence of current protocols for the surgical procedures and postoperative treatments resulted in a heterogeneous study population. Even though we included almost all patients treated with gentamicin-loaded CaS-HA biocomposite, and therefore obtained a representative study population for current clinical practice in the Netherlands, the resulting heterogeneity introduces various confounding factors which limit the conclusions that can be drawn. Third, the included study population was relatively small. Even though it is larger than in most previous studies, the small study population increases statistical errors. Therefore, the analyses performed should be considered exploratory and be interpreted cautiously. Fourth, our study is limited by the lack of a control group. In future prospective studies, a uniformed treatment regimen should be compared to a control cohort of patients treated by conventional treatments. Fifth, adequate investigation of ulcer recurrence was limited by the median follow-up of 43 weeks, which should ideally be 12 or 18 months after initial wound healing for this purpose [1]. Sixth, specific information regarding diabetes mellitus (i.e., glycemic control) and other risk factors for vascular disease (e.g., dyslipidemia or smoking status) was lacking, as well as details regarding the preoperative discontinuation of antibiotic therapy and the number and exact sites where bone samples were obtained. Seventh, we did not include a minimal postoperative duration in the definition of persistent wounds, which should be considered in further prospective studies. Eight, pre-operative functional status was not investigated in the assessment of functional results. Finally, assessment of persistent or recurrent osteomyelitis in patients with treatment failures was not possible, since additional investigations (e.g., imaging, bone samples) were not performed consistently. Future prospective studies will overcome these limitations.

Uniform treatment protocols are required for the treatment of DFO with gentamicin-loaded CaS-HA biocomposite. For a uniform treatment protocol, suggestions for patient selection should include patients with DFO after unsuccessful treatment by antibiotic therapy for at least 6 weeks [14]. Patients with severe limb ischemia should be excluded [33]. Regarding the surgical procedures, we advocate thorough surgical resection of DFO and obtaining multiple bone samples. Regarding postoperative treatments, we suggest offloading in accordance with the IWGDF guidelines until postoperative wound healing is observed, at least several days of postoperative antibiotic therapy based on the results of microbiological analysis of bone samples, and structural follow-up in a multidisciplinary setting [14,41]. Prospective investigation of treatment protocols, based on the insights reported in our study and previous studies, is required [23,24,28,29]. This prospective investigation should consider postoperative wound healing, ulcer recurrence, amputations and functional results in comparison to the pre-operative functional status.

In conclusion, surgical treatment with gentamicin-loaded CaS-HA biocomposite was feasible in this study of patients with DFO and successful in 66% of patients. A prospective trial of this treatment regimen, based on uniform treatment protocols, is required.

## Figures and Tables

**Table 1:** Study population characteristics stratified by outcome.

Characteristics	All Patients	Patients with Persistent Wounds	Patients with Ulcer Recurrence	Patients with Treatment Success	*p* Value
	(*N* = 64)	(*n* = 10)	(*n* = 12)	(*n* = 42)	
Follow-up in weeks	43 (20–61)	14 (12–27)	61 (23–91)	44 (28–61)	0.001 *
Male sex	50 (78)	8 (80)	9 (75)	33 (79)	>0.99
Age, years	63 ± 11	66 ± 9	56 ± 14	64 ± 11	0.089
Type of diabetes mellitus					
Type 1 diabetes	5 (8)	0	2 (17)	3 (7)	0.385
Type 2 diabetes	59 (92)	10 (100)	10 (83)	39 (93)	
Diabetes duration in years	17 (13–21)	17 (12–22)	16 (10–20)	17 (14–22)	0.359
Insulin treatment	40 (63)	5 (50)	9 (75)	26 (62)	0.482
Heart failure	18 (28)	3 (30)	3 (25)	12 (29)	>0.99
Nephropathy	21 (33)	4 (40)	6 (50)	11 (26)	0.262
Haemodialysis	5 (8)	0	2 (17)	3 (7)	0.385
BMI > 35 kg/m^2^	5 (8)	0	1 (8)	4 (10)	0.819
Retinopathy	23 (36)	4 (40)	4 (33)	15 (36)	>0.99
Charcot osteoarthropathy	18 (28)	0 (0)	7 (58)	11 (26)	0.010 **
Loss of protective sensation	59 (92)	10 (100)	12 (100)	37 (88)	0.496
Limb ischemia [33]					
None	33 (52)	3 (30)	10 (84)	20 (48)	0.035 ***
Mild	24 (37)	7 (70)	1 (8)	16 (38)	
Moderate	7 (11)	0	1 (8)	6 (14)	
Previous ipsilateral amputation					
None	42 (66)	9 (90)	6 (50)	27 (64)	0.486
Lesser toe	3 (5)	0	0	3 (7)	
Hallux/or single ray	9 (14)	1 (10)	4 (33)	4 (10)	
Multiple rays	6 (9)	0	1 (8)	5 (12)	
Forefoot	4 (6)	0	1 (8)	3 (7)	
Previous contralateral major amputation	4 (6)	0	1 (8)	3 (7)	>0.99
Foot deformity					
None	32 (50)	6 (60)	3 (25)	23 (55)	0.071
Mild	9 (14)	1 (10)	2 (17)	6 (14)	
Moderate	8 (13)	3 (30)	1 (8)	4 (10)	
Severe	15 (23)	0	6 (50)	9 (21)	
Previous ulcer on index ulcer location	24 (38)	5 (50)	7 (58)	12 (29)	0.111
Index ulcer duration in weeks	20 (13–46)	19 (10–42)	37 (21–79)	18 (10–43)	0.039 ^†^
Index ulcer location					
Forefoot	41 (64)	5 (50)	5 (42)	31 (74)	0.101
Midfoot	14 (22)	2 (20)	5 (42)	7 (17)	
Hindfoot	9 (14)	3 (30)	2 (17)	4 (9)	
Index ulcer on plantar aspect of foot	37 (58)	8 (80)	7 (58)	22 (52)	0.276
Index ulcer size in cm^2^	3 (2–5)	4 (3–4)	5 (2–7)	3 (2–4)	0.367
SINBAD classification score [34]	5 (4–5)	5 (4–5)	5 (4–5)	5 (5–5)	0.153
Previous treatment of DFO					
Surgical resection and antibiotic therapy	16 (25)	2 (20)	3 (25)	11 (26)	0.571
Minor amputation and antibiotic therapy	9 (14)	3 (30)	2 (17)	4 (10)	
Antibiotic therapy only	39 (61)	5 (50)	7 (58)	27 (64)	
Duration of antibiotic therapy in weeks	8 (6–10)	7 (6–10)	9 (6–19)	8 (6–10)	0.477
Preoperative revascularization procedure					
None	48 (75)	7 (70)	10 (83)	31 (74)	0.840
Endovascular procedure	15 (23)	3 (30)	2 (17)	10 (24)	
Antibiotic therapy only	1 (2)	0	0	1 (2)	
Surgical procedure with gentamicin-loaded CaS-HA biocomposite involved a minor amputation	8 (13)	4 (40)	3 (25)	1 (2)	0.001 ^††^
Fixation methods used in the surgical procedure with gentamicin-loaded CaS-HA biocomposite					
None	55 (86)	10 (100)	9 (75)	36 (86)	0.448
Internal fixation	7 (11)	0	2 (17)	5 (12)	
External fixation	2 (3)	0	1 (8)	1 (2)	
Microbiological analysis of osteomyelitis					
-Bone samples not obtained	15 (23)	3 (30)	2 (17)	10 (24)	0.755
-Monomicrobial infection	24 (38)	2 (20)	3 (25)	19 (45)	0.779
AGP pathogen	16 (25)	2 (20)	2 (17)	12 (28)	
AGN pathogen	8 (12)	0	1 (8)	7 (17)	
-Polymicrobial infection	25 (39)	5 (50)	7 (58)	13 (31)	0.179
AGP pathogens	11 (17)	1 (10)	2 (17)	8 (19)	
AGP pathogens and AGN pathogens	13 (20)	3 (30)	5 (42)	5 (12)	
AGP pathogens and OA pathogens	1 (2)	1 (10)	0	0	
-Gentamicin-resistant osteomyelitis	3 (5)	2 (20)	1 (8)	0	0.020 ^‡^

Data are presented as number (%), mean ± SD, or median (IQR). A *p*-value < 0.05 indicates significant differences between patient groups regarding the distribution of cases or reported values. For these variables, results of post hoc analyses with adjusted *p*-values by Bonferroni correction are presented in the footnote. SINBAD: Site, Ischemia, Neuropathy, Bacterial infection, Area, Depth classification. DFO: Diabetic foot ulcers complicated by osteomyelitis. CaS-HA: Calcium sulphate-hydroxyapatite. AGP: Aerobic, Gram-Positive. AGN: Aerobic Gram-negative. OA: Obligate anaerobic. * Postoperative follow-up, which was completed until amputation, repeated surgical resection of DFO, death, or the last-mentioned consultation in the electronic health record system, was significantly (*p* = 0.002) shorter in patients with persistent wounds. ** Distribution of Charcot osteoarthropathy was not significantly different between patient groups. *** Distribution of limb ischemia was not significantly different between patient groups. ^†^ Index ulcer duration was significantly longer in patients with ulcer recurrence (*p* = 0.014). ^††^ Of patients with persistent wounds, a significantly larger proportion underwent surgical procedures with gentamicin-loaded CaS-HA biocomposite that involved a minor amputation (*p* = 0.004). ^‡^ Of patients with persistent wounds, a significantly larger proportion had gentamicin-resistant DFO (*p* = 0.007).

**Table 2:** Univariable (*p* < 0.05) Cox regression analysis for treatment failures after surgical treatments of diabetic foot osteomyelitis with gentamicin-loaded calcium sulphate-hydroxyapatite biocomposite.

Characteristic	Univariable Analysis	
	Hazard Ratio (95%-CI)	*p* Value
Gentamicin-resistant osteomyelitis	3.847 (1065–13.899)	0.040
Index ulcer location		0.029
Forefoot	Reference	
Midfoot	3.022 (1127–8104)	0.028
Hindfoot	3.624 (1187–11.060)	0.024
Surgical procedure with gentamicin-loaded CaS-HA biocomposite involved a minor amputation	3.965 (1608–9777)	0.003

CI: Confidence interval. CaS-HA: Calcium sulphate-hydroxyapatite.

**Table 3:** Previous studies of treatments of diabetic foot osteomyelitis with gentamicin-loaded calcium sulphate-hydroxyapatite biocomposite.

Author (Year)	Study Design	Patients	Intervention	Follow-Up	Results	QUADAS-2 Score [37]
This study	Multicenter RCS	Inclusion of 64 patients with DFO after unsuccessful conventional treatment (antibiotic therapy alone, or surgical debridement or minor amputation with adjunctive antibiotic therapy).	Surgical debridement, dead space filling with gentamicin-loaded CaS-HA biocomposite, closure of skin and soft tissues. Procedures involved minor amputations in 8 patients (13%).	Median 43 (IQR, 20–61) weeks.	Wound healing in 54 patients (84%) and treatment success in 42 (66%).	Risk of bias:
			Postoperative offloading by non-weight bearing mobilization in 33 patients (52%), nonremovable knee-high devices in 14 (22%), removable knee-high devices in 2 (3%), and removable ankle-high devices in 15 (23%) for median 6 (IQR, 5–8) weeks.		Treatment failures (no wound healing) in 10 patients (12%).	Patient selection: Low risk
			Postoperative antibiotic therapy in 26 patients (41%) for a median 3 (IQR, 2–6) weeks.		Treatment failures (ulcer recurrence) in 12 patients (19%).	Index test: N/A
					Minor amputations in 4 patients (6%) and major amputations in 7 (11%) because of treatment failures.	Reference standard: N/A
					Weight-bearing mobilization at final follow-up in 50 patients (89%).	Flow and timing: Low risk
						Applicability concerns:
						Patient selection: Low risk
						Index test: N/A
						Reference standard: Low risk
Whisstock, et al. [29] (2020)	Single- center RCS	Inclusion of 35 patients (aged 18–80 years) with DFO, with or without Charcot neuroarthropathy and an otherwise normal function of the lower extremity.	Surgical debridement, dead space filling with gentamicin-loaded CaS-HA biocomposite. Procedures involved partial calcanectomies in 3 patients, talectomy in 1, and external fixation in 6 (17%).	12 months. Three patients lost to follow-up.	Bone infection cured in 26 patients (81%).	Risk of bias:
			Closure with dermal substitute (Hyalomatrix™) in 10 patients (29%).		Due to nonhealing, 1 minor and 3 major amputations were performed.	Patient selection: Low risk
			Postoperative antibiotic therapy for 4–6 weeks		Weight-bearing mobilization was possible in 25 patients (96%) with cured bone infections	Index test: N/A
			Postoperative offloading by total contact casts.			Reference standard: N/A
						Flow and timing: Low risk
						Applicability concerns:
						Patient selection: Low risk
						Index test: N/A
						Reference standard: Low risk
Hutting, et al. [28] (2019)	Case report	Treatment of 1 patient with CN-related deformity and midfoot DFO after unsuccessful surgical treatment.	Surgical debridement of DFO, dead space filling with gentamicin-loaded CaS-HA biocomposite, primary closure of skin and soft tissues.	12 months	Wound healing after 4 months.	Risk of bias:
					No ulcer recurrence during follow-up.	Patient selection: Unclear
			Enteral amoxicillin/clavulanate for 4 months.		Able to mobilize weight-bearing.	Index test: N/A
						Reference standard: N/A
						Flow and timing: Unclear
						Applicability concerns:
						Patient selection: Low risk
						Index test: N/A
						Reference standard: Low risk
Niazi, et al. [23] (2019)	Multicenter RCS	Inclusion of 70 patients with DFO of the forefoot (62%), midfoot (33%), or hindfoot (5%). CN-related deformity in 9 patients (13%)	Surgical debridement of DFO, dead space filling with gentamicin-loaded CaS-HA biocomposite (using the “Silo technique” in case of calcaneal DFO) [24], primary closure of skin and soft tissues or VAC. Procedures involved minor amputations in 2 patients (3%).	Mean 10 (range, 4–28) months	Wound healing in 57 patients (81%) after a mean of 12 (range, 4–16) weeks.	Risk of bias:
			Antibiotic therapy for mean 4 (range, 2–6) weeks.		Eradication of infection in 63 patients (90%).	Patient selection: High risk
					Treatment failures in 7 patients (10%).	Index test: N/A
					Major amputations in 5 patients (7%) due to treatment failures.	Reference standard: N/A
					No recurrence of infection.	Flow and timing: High risk
						Applicability concerns:
						Patient selection: Low risk
						Index test: N/A
						Reference standard: Low risk
Drampalos, et al. [24] (2018)	Single-center RCS	Inclusion of 12 patients with calcaneal DFO without involvement of the posterior subtalar joint.	Surgical resection, filling of drilled tunnels in the calcaneus with gentamicin-loaded CaS-HA biocomposite (“Silo technique”), primary closure or VAC.	Mean 16 (range, 12–18) weeks	Wound healing in 12 patients (100%) after mean 16 (range, 12–18) weeks.	Risk of bias:
			Antibiotic therapy for 6–12 weeks.		Postoperative ambulation in 6 patients.	Patient selection: High risk
						Index test: N/A
						Reference standard: N/A
						Flow and timing: High risk
						Applicability concerns:
						Patient selection: Low risk
						Index test: N/A
						Reference standard: Low risk

RCS: Retrospective cohort study. IQR: Interquartile range. CN: Charcot neuroarthropathy. DFO: Diabetic foot osteomyelitis. CaS-HA: Calcium sulphate–hydroxyapatite. VAC: Vacuum assisted closure. N/A: Not applicable.

## Data Availability

The data presented in this study are available on request from the corresponding author. The data are not publicly available due to confidentiality agreements. Supporting data can only be made available to bona fide researchers subject to a non-disclosure agreement. Details of the data and how to request access are available from Kor H. Hutting [ko.hutting@zgt.nl; 003188-7084232] at Hospital Group Twente, Department of Surgery, Zilvermeeuw 1, 7609 PP Almelo, the Netherlands.

## Appendix A

**Table A1:** Surgical procedures with gentamicin-loaded calcium sulphate-hydroxyapatite biocomposite performed to treat diabetic foot ulcers complicated by osteomyelitis in this study.

Surgical Procedures	No. (%)
Osteotomy of phalanx/phalanges, debridement and dead space filling with gentamicin-loaded CaS-HA biocomposite.	3 (5)
Arthrotomy of interphalangeal joint(s), resection with base and head of adjacent phalanges, dead space filling with gentamicin-loaded CaS-HA biocomposite.	3 (5)
Arthrotomy of interphalangeal joint(s), resection with base and head of adjacent phalanges, dead space filling with gentamicin-loaded CaS-HA biocomposite, arthrodesis with Kirschner wire.	3 (5)
Arthrotomy of metatarsophalangeal joint(s), resection with metatarsal head(s) and base of proximal phalanx/phalanges, dead space filling with gentamicin-loaded CaS-HA biocomposite.	14 (22)
Arthrotomy of metatarsophalangeal joint(s), resection with metatarsal head(s) and base of proximal phalanx/phalanges, dead space filling with gentamicin-loaded CaS-HA biocomposite, arthrodesis with Kirschner wire.	2 (3)
Osteotomy of metatarsal(s), debridement of medullary canal(s), filling with gentamicin-loaded CaS-HA biocomposite.	10 (16)
Amputation of metatarsal head(s), debridement of medullary canal(s) and filling with gentamicin-loaded CaS-HA biocomposite.	8 (13)
Arthrotomy of tarsometatarsal joint(s), resection of joint with distal part of one or multiple tarsals and base of one or multiple metatarsals, dead space filling with gentamicin-loaded CaS-HA biocomposite.	8 (13)
Arthrotomy of tarsometatarsal joint(s), resection with distal part of tarsal(s) and base of metatarsal(s), dead space filling with gentamicin-loaded CaS-HA biocomposite, temporary external fixation.	1 (2)
Arthrotomy of tarsometatarsal joint(s), resection with distal part of tarsal(s) and base of metatarsal(s), dead space filling with gentamicin-loaded CaS-HA biocomposite, internal screw fixation.	1 (2)
Partial or complete extirpation of tarsal(s), dead space filling with gentamicin-loaded CaS-HA biocomposite.	1 (2)
Partial or complete extirpation of tarsal(s), dead space filling with gentamicin-loaded CaS-HA biocomposite, temporary external fixation	2 (3)
Partial or complete extirpation of tarsal(s), dead space filling with gentamicin-loaded CaS-HA biocomposite, internal screw fixation.	1 (2)
Partial calcanectomy, surgical debridement and dead space filling with gentamicin-loaded CaS-HA biocomposite.	4 (6)
Partial calcanectomy, drilling of multiple tunnels in the calcaneum, filling with gentamicin-loaded CaS-HA biocomposite (“silo technique”) [24].	3 (5)
Treatment Characteristics	No. (%)
Volume of gentamicin-loaded CaS-HA biocomposite used (mL).	5 (4-5)
Form of gentamicin-loaded CaS-HA biocomposite used	
- Fluid phase	59 (92)
- Solid phase (e.g., pellets)	5 (8)
Use of pressure tourniquet during surgical procedure.	21 (33)
Method of surgical wound closure	
- Primary closure	57 (89)
- Local transposition	6 (9)
- Regional tissue transposition	1 (2)
Perioperative antibiotic therapy	
- None	32 (50)
- Carbapenems	1 (3)
- Cephalosporins	7 (23)
- Cephalosporins + trimethoprim/sulfamethoxazole	1 (3)
- Cephalosporins + fluoroquinolones	1 (3)
- Cephalosporins + macrolides	2 (6)
- Cephalosporins + metronidazole	1 (3)
- Fluoroquinolones + macrolides	5 (16)
- Penicillins	11 (34)
- Penicillins + fluoroquinolones	1 (3)
- Trimethoprim/sulfamethoxazole41	1 (3)
- Vancomycin	1 (3)
Route of administration of perioperative antibiotic therapy	
- Oral	6 (9)
- Parenteral	26 (41)
Method of anaesthesia	
- General	18 (28)
- Spinal	15 (24)
- Regional	29 (45)
- Local	2 (3)
Duration of surgery (minutes)	46 ± 21

Data are presented as number (%), mean ± SD or median (IQR). CaS-HA: Calcium sulphate-hydroxyapatite.
